# Determinants of quality of life in older adults with atrial fibrillation: a structural equation modeling analysis

**DOI:** 10.3389/fpubh.2026.1753021

**Published:** 2026-03-11

**Authors:** Chun-Yan Qiao, Yi Xu, Rong Jiang, Meng Zhang, Bi-Jun Huang, Lan Ding

**Affiliations:** Department of Cardiology, The Second Affiliated Hospital of Nanchang University, Nanchang, China

**Keywords:** anxiety, atrial fibrillation, depression, quality of life, social support, structural equation modeling

## Abstract

**Objective:**

This study aimed to examine the determinants that influence quality of life among older adults diagnosed with atrial fibrillation (AF) and to explore the direct and indirect pathways between these factors using structural equation modeling (SEM).

**Methods:**

A convenience sample of 252 older adults with AF who were admitted to the hospital between August 2023 and August 2024 was included. Data were collected using the Atrial Fibrillation Effect on Quality-of-Life (AFEQT) questionnaire, Self-Rating Depression Scale (SDS), Self-Rating Anxiety Scale (SAS), and Social Support Rating Scale (SSRS). Patients were categorized into high-quality (*n* = 158) and low-quality (*n* = 94) groups according to theira AFEQT scores. Independent determinants were identified through univariate analysis and multivariate logistic regression, followed by the construction of an SEM model to assess interrelationships among variables.

**Results:**

Age, monthly income, AF type, New York Heart Association functional classification, treatment adherence, SDS score, SSRS score, and presence of comorbid chronic conditions were identified as independent determinants of quality of life (*p* < 0.05). SEM demonstrated that age, monthly income, AF type, SDS score, and SSRS score exerted both direct effects (path coefficients of 0.200, −0.131, 0.134, 0.160, and −0.207, respectively) and indirect effects through mediating variables, resulting in total effect coefficients of 0.316, −0.168, 0.188, 0.225, and −0.347, respectively.

**Conclusion:**

Quality of life among older adults with AF was influenced by multiple interrelated factors. Social support, psychological status, and economic circumstances exerted significant combined effects through both direct and indirect pathways. The development of individualized, evidence-based nursing interventions that address these determinants may contribute to improved quality of life and overall health outcomes in this population.

## 1 Introduction

Atrial fibrillation (AF) is among the most prevalent cardiac arrhythmias encountered in clinical practice. It is characterized by disorganized atrial electrical activity that leads to the loss of effective atrial contraction and an irregular ventricular response. Clinical manifestations typically include palpitations, reduced exercise tolerance, heart failure, and significantly diminished quality of life (1).

Epidemiological data indicate a continuous increase in the global prevalence of AF, with older adults experiencing the highest disease burden. The prevalence of AF has been estimated to exceed 4% among individuals aged 65 years and older and to reach approximately 8–10% among those aged 80 years and above (2). In addition to elevating the risk of ischemic stroke and systemic embolism, AF contributes to increased hospital readmission rates and all-cause mortality, imposing a considerable healthcare and socioeconomic burden on both affected individuals and society (3).

In recent years, numerous studies have demonstrated that older adults diagnosed with AF generally experience a lower quality of life compared with those of age-matched healthy individuals or those with other chronic conditions (4). The determinants of quality of life in this population are multifactorial, encompassing biomedical, psychosocial, and treatment-related domains. From a biomedical perspective, factors such as heart failure, multimorbidity, AF type, and symptom burden have been strongly associated with reduced physiological functioning and diminished perceived wellbeing (5). Psychosocially, symptoms of anxiety and depression are prevalent, while insufficient social support and limited disease-related knowledge further impede coping and adaptive capacity (6).

Treatment-related factors, including therapeutic modality, adverse drug reactions, hemorrhagic risks associated with anticoagulant therapy, and the logistical burden of follow-up care, also negatively affect daily functioning to varying degrees (7). However, prior investigations have pre-dominantly examined single determinants or a narrow range of variables, lacking an integrated analysis of the complex interrelationships among multidimensional factors. This limitation has hindered the elucidation of underlying pathways and the estimation of effect sizes influencing quality of life, thereby constraining the formulation of targeted and effective intervention strategies. Structural equation modeling (SEM) is a multivariate statistical approach that integrates factor analysis and path analysis, allowing for the simultaneous examination of both manifest and latent variables. This method facilitates the evaluation of complex direct, indirect, and total effects among multiple interrelated variables. SEM is particularly suitable for analyzing the multilevel and interactive influences of biopsychosocial factors on health outcomes (8).

In the present study, older adults diagnosed with AF who received inpatient care at the study institution were included. Multidimensional data were obtained through a cross-sectional survey. The study objectives were to (1) determine the independent factors associated with quality of life; (2) construct and validate a structural equation model to elucidate the pathways and strengths of the relationships among variables; and (3) generate empirical evidence to support the development of comprehensive nursing intervention strategies aimed at improving quality of life. The results were intended to address existing gaps in multifactorial analyses of quality of life in this population and to provide a theoretical foundation for the implementation of personalized and holistic healthcare approaches.

The comprehensive geriatric assessment (CGA) provides a structured framework for evaluating the multidimensional health status of older patients, encompassing physical, functional, cognitive, psychological, and social domains. In the context of AF, a holistic assessment that includes CGA components is crucial for understanding the complex determinants of quality of life. Recent observational studies have begun to incorporate such comprehensive assessments in hospitalized elderly populations to evaluate prognostic associations. Notably, emerging evidence suggests that sex-based differences may play a significant role in the clinical presentation, psychosocial burden, and outcomes of older adults with AF, warranting further exploration in multifactorial models (9).

Furthermore, physical function, a core component of the comprehensive geriatric assessment, is frequently impaired in hospitalized elderly patients with AF and is a strong predictor of clinical outcomes and quality of life. The Short Physical Performance Battery (SPPB) is a validated tool for objectively assessing lower extremity function, and its prognostic value in this specific population has been recently underscored (10).

## 2 Materials and methods

### 2.1 Study participants

The sample size was determined in accordance with the methodological requirements for SEM (6). The number of variables included in the factor analysis was first identified, comprising 16 factors in total. According to standard recommendations, the sample size should be 10 to 15 times the number of variables, yielding a target range of 160–240 participants. To enhance model stability and ensure estimation precision, the maximum recommended sample size of 240 was adopted. Considering an anticipated attrition rate of 10%, the final required sample size was calculated as 264 participants.

Based on the number of older adults diagnosed with AF who received treatment at the study institution, a total of 252 patients were recruited through convenience sampling between August 2023 and August 2024.

**Inclusion criteria:** (1) A diagnosis of AF confirmed by the cardiology department in accordance with the *American College of Cardiology/European Society of Cardiology/American Heart Association* guidelines for the management of atrial fibrillation (7), classifying AF as paroxysmal, persistent, or permanent. (2) Age > 60 years, with complete and verifiable clinical data. (3) Ability to complete the questionnaire assessments independently or with assistance. (4) Provision of voluntary informed consent.

**Exclusion criteria:** (1) Presence of hepatic failure, cerebral dysfunction, renal failure, or malignant tumors. (2) Presence of congenital intellectual disability or clinically diagnosed cognitive impairment.

### 2.2 Factor analysis indicators

#### 2.2.1 Quality of life assessment

During hospitalization, the quality of life among patients was assessed using the Atrial Fibrillation Effect on Quality-of-Life (AFEQT) questionnaire (8). This instrument was specifically developed to evaluate quality of life in patients diagnosed with AF and comprises four domains: symptoms, mental health, physical functioning, and social activities. The questionnaire includes 20 items, each scored on a scale yielding a total score ranging from 0 to 100. Higher scores reflect better perceived quality of life. A total score of ≥75 points was considered indicative of good quality of life, whereas a score < 75 points denoted a moderate or lower level of quality of life.

#### 2.2.2 General data collection

Clinical and demographic data were obtained during hospitalization. Collected variables included patient age, sex, smoking history, alcohol consumption history, marital status, educational level, average monthly per capita household income, AF type, New York Heart Association (NYHA) functional classification, and the presence of comorbid chronic conditions.

#### 2.2.3 Scale data collection

During hospitalization, multiple standardized assessment instruments were administered to collect scale data, as described below:

**Warfarin anticoagulation compliance scale** (11): this instrument was used to assess adherence to anticoagulation therapy among patients diagnosed with AF. It includes questions evaluating the patients' understanding of medication use, consistency in dosing schedules, frequency of missed doses, and awareness of potential adverse drug reactions as well as associated risks. The total score ranges from 0 to 20, with higher scores indicating better adherence to anticoagulant therapy.

**Self-management ability assessment scale** (12): this scale was employed to evaluate self-management ability among patients with AF receiving warfarin therapy. It comprises 28 items across three dimensions: management of AF etiology and triggers, medication management, and control of disease severity. The total score is 84 points, with higher scores reflecting better self-management capabilities.

**Self-rating depression scale (SDS)** (13): this instrument measures the severity of depressive symptoms across emotional, cognitive, and somatic domains. It consists of 20 items, each rated on a four-point scale ranging from “almost never” to “most of the time.” Higher scores correspond to greater levels of depressive symptoms.

**Self-rating anxiety scale (SAS)** (14): this scale assesses the severity of anxiety, encompassing emotional, physiological, and cognitive aspects. It includes 20 items rated on a four-point scale from “never” to “almost all day”. Higher scores denote more severe anxiety symptoms.

**Social support rating scale (SSRS)** (15): this instrument evaluates the degree of perceived social support, incorporating three dimensions: subjective support, objective support, and support utilization. It consists of 10 items addressing support from family members, friends, and other sources. Each item is scored from 1 (strongly disagree) to 4 (strongly agree), yielding a total score ranging from 10 to 64 points. Higher scores indicate greater perceived social support.

**Pittsburgh sleep quality index (PSQI)** (16): this scale measures overall sleep quality and patterns. It includes 19 self-rated and five externally rated items assessing components such as subjective sleep quality, sleep latency, sleep duration, and sleep disturbances. Total scores range from 0 to 21, with higher scores indicating poorer sleep quality.

### 2.3 Statistical methods

Statistical analysis was conducted using SPSS 21.0 software (IBM Corp., Armonk, NY, USA). Continuous data were presented as mean ± standard deviation ($\bar{X}$ ± s), and inter-group comparisons were conducted using the independent-samples *t*-test. Categorical variables were expressed as frequencies and percentages [*n* (%)], and inter-group differences were assessed using the chi-squared test (χ^2^).

Binary logistic regression analysis was employed to identify the factors associated with quality of life, with a two-tailed *p-*value < 0.05 considered statistically significant. SEM was constructed using AMOS version 21.0 software to analyze the relationships among observed variables.

The model fit was considered acceptable when the chi-square value (χ^2^ statistic, CMIN) ranged from 1 and 3; the Adjusted Goodness-of-Fit Index (AGFI), Incremental Fit Index (IFI), Tucker-Lewis Index (TLI), and Comparative Fit Index (CFI) were all > 0.900; and the Root Mean Square Error of Approximation (RMSEA) was < 0.05. The significance level was set at α = 0.05.

## 3 Results

### 3.1 Analysis of quality of life in older adults with AF

Among the 252 older adults diagnosed with AF who received treatment at the study institution, 158 patients (62.70%) achieved AFEQT scores of ≥75 points, indicating a high level of quality of life. The remaining 94 patients (37.30%) had AFEQT scores of < 75 points, corresponding to a lower level of quality of life.

### 3.2 Univariate analysis of factors associated with quality of life in older adults with AF

Univariate analysis demonstrated no statistically significant differences between the high-and low-quality-of-life groups with respect to sex, smoking history, alcohol consumption history, marital status, educational level, or PSQI scores (*p* > 0.05). In contrast, significant differences were observed for age, average monthly per capita household income, AF type, NYHA functional classification, treatment adherence, SDS scores, SAS scores, SSRS scores, and the presence of comorbid chronic conditions (*p* < 0.05), as presented in Table 1.

**Table 1:** Univariate analysis of factors associated with quality of life among older adults with AF (*n*, $\bar{x}$ ± s).

**Influencing factors**	**Low quality of life group (*n* = 94)**	**High quality of life group (*n* = 158)**	***t*/χ^2^*/F***	***p***
Age (years)	73.18 ± 3.54	71.24 ± 3.28	4.408	< 0.001
Sex			0.451	0.502
Male	64 (68.09)	101 (63.92)		
Female	30 (31.91)	57 (36.08)		
Smoking history			1.026	0.311
Yes	43 (45.74)	62 (39.24)		
No	51 (54.26)	96 (60.76)		
Alcohol consumption history			1.225	0.268
Yes	46 (48.94)	66 (41.77)		
No	48 (51.06)	92 (58.23)		
Educational level			0.459	0.711
Primary school	14 (14.89)	31 (19.63)		
Junior high school	37 (39.36)	53 (33.54)		
Senior high school and technical secondary school	25 (26.60)	45 (28.48)		
Junior college and above	18 (19.15)	29 (18.35)		
Marital status			0.442	0.643
Married	77 (81.91)	122 (77.21)		
Unmarried	12 (12.77)	27 (17.09)		
Widowed	5 (5.32)	9 (5.70)		
Average monthly household income per capita			6.466	0.011
< RMB 5000	53 (56.38)	63 (39.87)		
≥RMB 5000	41 (43.62)	95 (60.13)		
AF type			6.569	0.010
Paroxysmal	55 (58.51)	117 (74.05)		
Persistent or permanent	39 (41.49)	41 (25.95)		
NYHA classification			0.451	0.502
Class II	34 (36.17)	79 (50.00)	4.558	0.033
Class III and IV	60 (63.83)	79 (50.00)		
Treatment adherence (score)	10.69 ± 1.21	11.35 ± 1.44	3.728	< 0.001
Self-management ability (score)	45.20 ± 5.37	46.17 ± 5.26	1.405	0.161
SDS score	36.42 ± 3.16	34.88 ± 3.01	3.855	< 0.001
SAS score	31.67 ± 2.70	30.73 ± 2.55	2.768	0.006
SSRS score	39.78 ± 5.42	42.71 ± 5.49	4.117	< 0.001
PSQI score	14.36 ± 1.53	13.97 ± 1.57	1.925	0.055
Comorbid chronic diseases			5.572	0.018
Yes	45 (47.87)	52 (32.91)		
No	49 (52.13)	106 (67.09)		

### 3.3 Multivariate logistic regression analysis of factors associated with quality of life in older adults with AF

Multivariate logistic regression analysis was conducted with quality of life as the dependent variable and the variables demonstrating significant differences in univariate analysis as independent predictors. Variable assignments are summarized in Table 2. The analysis indicated that age, AF type, NYHA functional classification, SDS scores, and the presence of comorbid chronic conditions were independent risk factors for lower quality of life among older adults with AF. In contrast, higher average monthly per capita household income, greater treatment adherence, and higher SSRS scores were identified as independent protective factors (*p* < 0.05), as presented in Table 3.

**Table 2:** Variable assignment for independent factors influencing quality of life among older adults with AF.

**Variable**	**Assignment**
Age	Raw data entry
Average monthly household income per capita	≤ 5000 CNY = 0, >5000 CNY = 1
AF type	Paroxysmal = 0, Persistent or permanent = 1
NYHA classification	Class II = 0, Class III and IV = 1
Treatment adherence	Raw data entry
SDS score	Raw data entry
SAS score	Raw data entry
SSRS score	Raw data entry
Comorbid chronic diseases	No = 0, Yes = 1

**Table 3:** Multivariate logistic regression analysis of factors influencing quality of life among older adults with AF.

**Related factors**	**β Coefficient**	**SE**	**Wald statistic**	**OR**	**95% CI**		***p*-value**
					**Lower limit**	**Upper limit**	
Age	0.182	0.049	13.507	1.199	1.089	1.321	< 0.001
Average monthly household income per capita	−0.777	0.318	5.977	0.460	0.247	0.857	0.014
AF type	0.668	0.337	3.928	1.951	1.007	3.778	0.047
NYHA classification	0.749	0.325	5.325	2.115	1.120	3.997	0.021
Treatment adherence	−0.425	0.121	12.284	0.654	0.515	0.829	< 0.001
SDS score	0.139	0.053	6.940	1.149	1.036	1.275	0.008
SAS score	0.105	0.060	3.007	1.110	0.986	1.250	0.083
SSRS score	−0.107	0.030	12.732	0.898	0.847	0.953	< 0.001
Comorbid chronic diseases	0.658	0.320	4.219	1.931	1.031	3.619	0.040

### 3.4 Construction of the SEM for quality of life in older adults with AF

A path diagram for the SEM was developed based on findings from the literature and the results of the multivariate logistic regression analysis. The regression analysis identified age, average monthly per capita household income, AF type, NYHA functional classification, treatment adherence, SDS scores, SSRS scores, and the presence of comorbid chronic conditions as significant factors associated with quality of life.

Existing studies further indicate that advanced age increases the likelihood of comorbid chronic conditions (17), AF type is correlated with NYHA functional classification (18), and social support influences depressive symptoms and treatment motivation. Additionally, both depressive symptoms and income level have been shown to affect treatment adherence. Based on these findings, a structural equation model was established. The model was refined iteratively according to theoretical considerations and modification indices until satisfactory parameter estimates were achieved.

**Figure 1:**
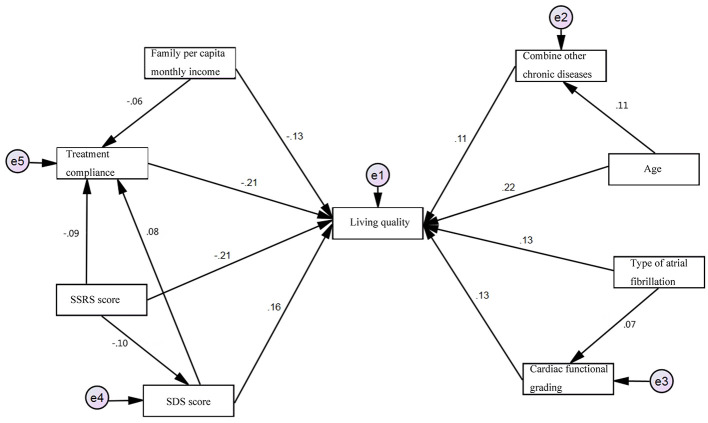
SEM of quality of life among older adults with AF.

Path coefficients are presented in Table 4, and the final SEM is illustrated in Figure 1. The analysis revealed that all eight independent variables exerted direct effects on patients' quality of life. Indirect pathways were also identified: age affected quality of life indirectly through its influence on comorbid chronic conditions; AF type affected quality of life indirectly through its association with NYHA classification; and monthly income, SSRS scores, and SDS scores influenced quality of life indirectly by affecting treatment adherence. Furthermore, SSRS scores had an additional indirect effect on quality of life through their impact on SDS scores. The coefficients of indirect effects are summarized in Table 5.

**Table 4:** SEM path coefficients for factors affecting quality of life.

**Path**	**Path coefficient**	**S. E**	**C.R**.	***p***
Quality of life ← age	0.220	0.007	3.978	< 0.001
Quality of life ← average monthly household income per capita	−0.131	0.052	−2.368	0.018
Quality of life ← AF type	0.134	0.056	2.429	0.015
Quality of life ← NYHA classification	0.125	0.053	2.268	0.023
Quality of life ← treatment adherence	−0.206	0.019	−3.728	< 0.001
Quality of life ← SDS score	0.160	0.008	2.906	0.004
Quality of life ← SSRS score	−0.207	0.005	−3.738	< 0.001
Quality of life ← comorbid chronic diseases	0.114	0.054	2.062	0.039
Comorbid chronic diseases ← age	0.105	0.005	3.253	0.018
NYHA classification ← AF type	0.069	0.017	1.747	0.034
Treatment adherence ← average monthly household income per capita	−0.058	0.012	−1.960	< 0.001
Treatment adherence ← SSRS score	−0.094	0.004	−3.141	0.026
SDS score ← SSRS score	−0.101	0.007	−2.975	0.042
Treatment adherence ← SDS score	0.077	0.005	2.493	0.035

**Table 5:** Indirect effect coefficients of factors influencing quality of life among older adults with AF.

**Influencing factors**	**Indirect effect**	**Path coefficient**	**Total path coefficient**	***p*-value**
Age	Age → comorbid chronic diseases → quality of life	0.096	0.316	0.018
AF type	AF type → NYHA classification → quality of life	0.054	0.188	0.006
Average monthly household income per capita	Average monthly household income per capita → treatment adherence → quality of life	−0.037	−0.168	0.022
SSRS score	SSRS score → treatment adherence, SDS score → quality of life	−0.140	−0.347	0.033
SDS score	SDS score → treatment adherence → quality of life	0.065	0.225	0.040

The final SEM for quality of life among older adults with AF demonstrated satisfactory model fit, with χ^2^ = 146.731, *p* < 0.001, CMIN = 1.317, AGFI = 0.951, IFI = 0.922, TLI = 0.850, CFI = 0.908, and RMSEA = 0.036, indicating a good fit between the model and the observed data.

## 4 Discussion

### 4.1 Current status of quality of life among older adults with AF

Multiple studies have indicated that the quality of life among older adults diagnosed with AF is generally below optimal levels. For instance, a web-based multicenter study conducted in the United States involving 295 patients with AF reported an average AFEQT score of 76.3, representing a moderate level of quality of life. Existing research further demonstrates that reduced quality of life adversely affects physical health, psychological wellbeing, daily functional capacity, and social interactions.

A lower quality of life has been associated with an increased psychological burden and exacerbation of clinical symptoms, including palpitations, dyspnea, and fatigue, which collectively limit daily activities and elevate the risk of adverse health outcomes, particularly in older adults. Understanding the determinants that contribute to diminished quality of life in this population and clarifying their interrelationships through SEM provides a foundation for developing targeted, evidence-based nursing interventions. Such interventions are expected to support improved health outcomes and overall wellbeing among older adults with AF.

### 4.2 Analysis of influencing factors of quality of life

#### 4.2.1 Direct path influencing factors of quality of life

Results from the SEM demonstrated that age, average monthly per capita household income, AF type, NYHA classification, treatment adherence, SDS scores, SSRS scores, and the presence of comorbid chronic conditions exerted direct effects on the quality of life among older adults diagnosed with AF.

The potential mechanisms underlying these associations are multifactorial. Advancing age is accompanied by progressive declines in physiological function across multiple systems, including the cardiovascular, immune, and metabolic systems. This age-related deterioration contributes to reduced resilience and poorer overall quality of life. In contrast, higher household income levels are associated with improved living conditions, including better nutrition, safer housing environments, and enhanced access to healthcare and social activities, all of which positively influence both physical and psychological wellbeing.

Variations in AF type also contribute to differential symptom burdens. Persistent and permanent AF are typically associated with more pronounced clinical manifestations, such as palpitations, chest pain, and syncope, compared to paroxysmal AF, thereby exerting a more substantial negative impact on perceived comfort and quality of life. Additionally, the NYHA functional classification, which reflects the degree of impairment in cardiac performance, directly influences physical endurance and capacity for daily activities. Higher NYHA classes, indicative of more severe cardiac dysfunction, are correlated with greater functional limitations and consequently lower quality of life.

Good treatment adherence facilitates consistent pharmacologic and non-pharmacologic management of AF, enabling effective control of cardiac rhythm disturbances and reducing the risk of complications such as thromboembolism. This stability in disease management contributes to improved physical health and, consequently, enhances overall quality of life.

Depressive symptoms are frequently associated with diminished motivation, emotional withdrawal, and reduced engagement in disease management among older adults with AF. These psychological manifestations contribute to reduced life satisfaction and an overall decline in quality of life.

Social support, as quantified by the SSRS, represents the level of emotional, informational, and practical assistance available from family members, peers, and the broader social network. Higher levels of social support facilitate greater understanding, care, and assistance in daily living, which collectively mitigate psychological distress and feelings of loneliness, thereby enhancing life satisfaction and overall quality of life. Older adults with AF frequently present with comorbid chronic conditions such as hypertension and chronic obstructive pulmonary disease. These comorbidities are often accompanied by physical discomfort, including headaches, chest pain, and excessive fatigue. In addition, functional limitations such as decreased mobility and visual impairment further restrict daily activities and negatively affect perceived quality of life in this population.

#### 4.2.2 Indirect path influencing factors of quality of life

SEM results indicated that age, AF type, average per capita monthly household income, SSRS scores, and SDS scores exerted indirect effects on quality of life among older adults diagnosed with AF.

Several mechanisms may account for these indirect pathways. With advancing age, progressive physiological decline across multiple organ systems increases susceptibility to comorbid chronic conditions. The coexistence and interaction of such conditions, including cardiovascular, metabolic, and respiratory disorders, often exacerbate disease severity and contribute to a deterioration in overall quality of life. Furthermore, a close association has been observed between NYHA functional classification and AF type. As AF evolves from paroxysmal to persistent and permanent forms, cardiac function may progressively decline, leading to higher NYHA classifications and corresponding reductions in exercise tolerance, thereby negatively affecting quality of life.

Socioeconomic factors also play a significant role. Patients from lower-income households frequently encounter financial constraints that limit access to healthcare resources, including prescribed medications, diagnostic assessments, and interventional procedures. Such limitations may result in irregular medication use or missed medical appointments, which subsequently reduce treatment adherence and adversely affect disease management and quality of life.

Higher levels of social support, as reflected by SSRS scores, provide emotional, informational, and practical assistance during disease management. Strong family and community support enhance adherence to therapeutic regimens, promote psychological stability, and facilitate better coping mechanisms, all of which contribute to improved quality of life. In addition, social support fosters psychological resilience and adaptive capacity, reducing susceptibility to depressive symptoms and promoting a more positive outlook on illness and recovery.

Conversely, elevated SDS scores, indicating more severe depressive symptoms, are often associated with diminished motivation, reduced trust in medical care, and lower adherence to treatment recommendations. These psychological barriers interfere with consistent disease management and contribute to suboptimal treatment outcomes, ultimately reducing quality of life among older adults with AF.

#### 4.2.3 The role of sex differences in quality of life

While the current SEM did not identify sex as a statistically significant factor in univariate analysis, this finding should be interpreted with caution. Our sample may have had limited power to detect sex-specific effects. Importantly, recent comprehensive assessments in hospitalized elderly AF patients highlight significant sex-based differences. A study by Armentaro et al. (9) utilizing a CGA found distinct profiles between older men and women with non-valvular AF. Women in their study exhibited a higher prevalence of physical function impairment, depressive symptoms, and poorer nutritional status compared to men, despite similar ages and cardiovascular risk profiles (9). These CGA domains are closely linked to quality of life. The observed female pre-ponderance in depressive symptoms and functional limitations aligns with broader psychosocial literature and suggests potential indirect pathways through which sex may influence quality of life in AF, possibly mediated by psychological distress (SDS) and physical functioning (reflected in NYHA class or comorbidities). Our model identified SDS scores and comorbidities as significant direct and indirect factors, which are domains where women in the cited study showed greater vulnerability. Future research with larger samples should explicitly incorporate sex as a potential moderating variable in the pathways between psychosocial factors, comorbidities, and quality of life, as tailoring interventions based on sex-specific vulnerabilities (e.g., enhanced psychological support for women, focused functional rehabilitation) could be a crucial step toward personalized care.

#### 4.2.4 The prognostic role of physical function

Our study assessed general health status and NYHA class but did not include a specific, objective measure of physical performance. This represents a potential limitation, as functional capacity is a key determinant of independence and quality of life in the elderly. Recent research highlights the critical importance of such assessments. A study by Armentaro et al. (10) demonstrated that the SPPB score is an independent predictor of all-cause mortality and rehospitalization in elderly patients hospitalized with AF, even after adjusting for conventional risk factors. This finding underscores that objective physical function provides prognostic information beyond subjective symptom reporting (like NYHA class) or the presence of comorbidities. Poor SPPB performance, indicative of sarcopenia, frailty, and balance deficits, likely influences quality of life through multiple pathways: by directly limiting activities of daily living and social participation; by increasing fear of falls and reducing self-efficacy; and by interacting with psychological factors such as depression. In our model, pathways involving comorbidities and NYHA class may partially capture the influence of physical decline, but a direct measure like SPPB could refine the model. Future studies investigating quality of life in older AF patients should incorporate objective functional assessments like the SPPB to better elucidate this crucial dimension and identify patients who may benefit from targeted rehabilitative interventions.

#### 4.2.5 Considerations regarding functional assessment: NYHA vs. European heart rhythm association (EHRA) classification

In this study, we utilized the NYHA functional classification to assess the impact of cardiac-related symptoms on patients' daily activities and quality of life. While NYHA classification is a well-established and widely used tool for grading the severity of heart failure symptoms and activity limitation, it is recognized that not all patients with AF have concomitant clinical heart failure. The rationale for its inclusion was based on the high prevalence of overlapping symptoms (e.g., dyspnea, fatigue, exercise intolerance) between AF and heart failure, and its strong demonstrated association with quality of life and prognosis in cardiovascular populations. Furthermore, NYHA class serves as a practical, clinician-friendly indicator of overall functional disability attributable to cardiac conditions.

However, the EHRA symptom classification is a more AF-specific tool designed to quantify the impact of AF-related symptoms themselves on daily life and quality of life, ranging from EHRA I (no symptoms) to EHRA IV (severe symptoms disabling daily activities). It may offer a more direct assessment of the symptom burden attributable specifically to the arrhythmia, independent of left ventricular function. Our use of NYHA, while providing valuable prognostic and functional information, may not have fully captured the nuanced spectrum of AF-specific symptom severity. Future studies aiming to dissect the precise mechanisms linking arrhythmia burden to quality of life could benefit from incorporating both NYHA and EHRA classifications to differentiate the contributions of general cardiac dysfunction from those of AF-specific symptoms. This would allow for a more granular analysis and potentially more targeted symptom management strategies.

### 4.3 SEM analysis results

All goodness-of-fit indices for the SEM demonstrated satisfactory values, indicating that the model appropriately represented the interrelationships among factors influencing quality of life. The model outcomes provide a scientific basis for the development of targeted nursing interventions aimed at improving the health-related quality of life among older adults diagnosed with AF.

Educational interventions may be implemented through structured face-to-face sessions, during which comprehensive and accessible information regarding AF can be delivered. Such programs may address the etiology of AF, therapeutic objectives, available treatment modalities, and the significance of adherence to prescribed therapy. Providing education in this format facilitates better understanding of the disease process and promotes active engagement in disease management.

Family involvement and the use of digital communication platforms can strengthen continuity of care and social support for older adults diagnosed with AF. Establishing online communication channels, such as WeChat groups, enables healthcare professionals to disseminate educational materials, provide ongoing guidance, and supply psychosocial support resources. Family members can be encouraged to monitor treatment adherence and health status, thereby reinforcing social support and contributing to improved treatment compliance.

Psychological interventions play an essential role in managing emotional disturbances associated with AF. Physicians and mental health professionals can employ evidence-based approaches such as cognitive behavioral therapy, eye movement desensitization and reprocessing, and structured counseling to address anxiety, fear, and depressive symptoms. When clinically indicated, pharmacologic therapy may be considered, including the use of antidepressant agents such as paroxetine, amitriptyline, or mirtazapine, in accordance with individual tolerance and clinical guidelines.

For patients with comorbid chronic conditions, concurrent pharmacologic management should be optimized to control underlying diseases while maintaining effective AF treatment. In cases of severe or treatment-refractory AF, interventional procedures such as catheter ablation or pacemaker implantation may be considered to restore and maintain normal cardiac rhythm.

For older adults with limited financial resources, individualized dietary and lifestyle interventions are also recommended. Nutritional management should emphasize a balanced diet with reduced intake of high-fat, high-sodium, and high-sugar foods, along with increased consumption of fresh fruits, vegetables, and whole grains. Regular, moderate aerobic exercise tailored to the individual's physical capacity can support cardiovascular and respiratory functions, thereby promoting overall health and enhancing quality of life.

### 4.4 Limitations

This study has several limitations. The sample size was relatively small, and all participants were recruited from a single hospital, which restricts the representativeness and external validity of the findings. Additionally, variables such as health literacy and living environment were not assessed due to limited research resources. Further investigation through multicenter studies with larger and more diverse cohorts is warranted to validate these findings and enhance their generalizability. Furthermore, consideration of patient sex, as highlighted in recent CGA-based studies, may uncover additional nuances in vulnerability profiles (e.g., higher depressive symptoms and functional impairment in women) that warrant attention in personalized care planning (9). Additionally, the integration of objective physical function assessments, such as the Short Physical Performance Battery, into the routine evaluation of elderly hospitalized AF patients could provide valuable prognostic information and identify a critical modifiable target for interventions aimed at preserving independence and improving quality of life (10).

## 5 Conclusion

The quality of life among older adults diagnosed with AF is influenced by multiple interrelated factors, such as age, AF type, and the level of social support. Specifically, age, average monthly household income, AF type, SDS scores, and SSRS scores exert both direct and indirect effects on quality of life. These variables may assist healthcare professionals in identifying patients most adversely affected and in developing individualized nursing interventions aimed at improving quality of life and promoting both physical and psychological wellbeing.

## Data Availability

The original contributions presented in the study are included in the article/supplementary material, further inquiries can be directed to the corresponding author.
